# Imported Zika Virus Infection from the Cook Islands into Australia, 2014

**DOI:** 10.1371/currents.outbreaks.4635a54dbffba2156fb2fd76dc49f65e

**Published:** 2014-06-02

**Authors:** Alyssa T. Pyke, Michelle T. Daly, Jane N. Cameron, Peter R. Moore, Carmel T. Taylor, Glen R. Hewitson, Jan L. Humphreys, Richard Gair

**Affiliations:** Forensic and Scientific Services, Coopers Plains, Queensland, Australia; Tropical Public Health, Townsville, Australia; Forensic and Scientific Services, Coopers Plains, Queensland, Australia; Forensic and Scientific Services, Coopers Plains, Queensland, Australia; Forensic and Scientific Services, Coopers Plains, Queensland, Australia; Forensic and Scientific Services, Coopers Plains, Queensland, Australia; Tropical Public Health, Townsville, Australia; Tropical Public Health, Cairns, Australia

**Keywords:** arbovirus, infectious disease

## Abstract

A female resident of Townsville, Queensland, Australia has been diagnosed with Zika virus infection following a recent trip to the Cook Islands. An initial serum sample collected in March, 2014 was positive by two separate Zika virus TaqMan real-time RT-PCRs and a pan-Flavivirus RT-PCR. Nucleotide sequencing and phylogenetics of the complete Cook Islands Zika virus envelope gene revealed 99.1% homology with a previous Cambodia 2010 sequence within the Asian lineage. In addition, IgG and IgM antibody seroconversions were detected between paired acute and convalescent phase sera using recombinant Zika virus serology assays. This is the first known imported case of Zika virus infection into northern Queensland where the potential mosquito vector <i>Aedes aegypti</i> is present and only the second such reported case diagnosed within Australia.

## Introduction

A member of the family *Flaviviridae*, genus *Flavivirus*, Zika virus (ZIKV) is a mosquito-borne pathogen of medical significance which, like dengue virus (DENV), has caused outbreaks of human infection in Africa, Asia and the Pacific region. Natural transmission and onset of human disease follows the bite of an infected mosquito and predominantly involves the *Aedes* species, including the domestic vector *Aedes aegypti* 1. Primarily maintained in a zoonotic cycle involving non-human primates, ZIKV was first isolated in 1947 from a febrile rhesus monkey placed as a sentinel animal in the Zika Forest, Uganda^2^. Alternatively, humans may be implicated as amplification and reservoir hosts in regions not inhabited by non-human primates^3^.

Typically, clinical manifestations of human ZIKV disease are similar to many arboviral infections that occur without serious complications and may include a self-limiting febrile illness, arthralgia, myalgia, headache and maculopapular rash^4^^,^ 5 ^,^ 6. Conjuctivitis, retro-orbital eye pain, lymphadenopathy and diarrhoea have also been reported^4^, ^7^. However, in comparison with the diagnosis of infections caused by the more prevalent arboviruses DENV and chikungunya virus (CHIKV), many clinicians and laboratories are not yet familiar with ZIKV and consequently may miss or incorrectly diagnose ZIKV infections^3^.

Within Southeast Asia, distribution of human ZIKV infection has been found in several countries including Thailand, Vietnam and Malaysia. Seroprevalence in Lombok and Indonesia has also been reported revealing evidence of human neutralising antibodies^1^. In 2007, an unprecedented outbreak of ZIKV occurred on Yap Island, Micronesia, most probably originating from a Southeast Asian strain^3^^,^ 4. More recently, ZIKV has been rapidly spreading unchecked across the South Pacific since 2013 causing epidemics in French Polynesia, New Caledonia, Easter Island and the Cook Islands^8^. Although no local transmission has been recorded in Australia, the close proximity of ZIKV activity in surrounding regions and increased importation of related arboviruses such as DENV and CHIKV via viraemic travelers potentially compounds disease detection and constitutes a major public health concern. Additionally, *Aedes aegypti* mosquitoes are currently only found in Queensland and the Torres Strait which are already under regular threat from sporadic DENV outbreaks. Another implicated ZIKV mosquito vector, *Aedes albopictus* has been identified in the Torres Strait and could potentially become established on the mainland^9^. Importation of at least one case of ZIKV into Australia has been previously reported^5^. Here we describe the first reported case and diagnosis of ZIKV infection from a returned traveler into Townsville, north Queensland from the Cook Islands

## Materials and Methods

Acute and convalescent serum samples collected 8 days apart from a 65 year old female were tested at Forensic and Scientific Services (FSS), Queensland. Patient information provided on acute sample submission included 2 days of illness with symptoms of rash, lethargy, nausea and joint pain and history of recent travel to the Cook Islands in March, 2014. Suspected as a possible ZIKV infection, several assays were employed to attempt diagnosis of the illness including routine flavivirus screening. Of note, we were able to trial in-house molecular and serological ZIKV assays which were developed to potentially enhance laboratory capability and provide rapid and specific testing until live ZIKV stocks and positive patient control samples can be obtained. Molecular assays included two specific ZIKV real-time RT-PCRs designed in the envelope (E) and nonstructural protein 1 (NS1) genes respectively: ZIKV-E, Zika E For Primer: 5’-1222AAGTTTGCATGCTCCAAGAAAAT1244-3’, Zika E Probe: 5’-FAM-1246ACCGGGAAGAGCATCCAGCCAGA1268-TAMRA-3’, Zika E Rev Primer: 5’-1293CAGCATTATCCGGTACTCCAGAT1271-3’ and ZIKV-NS1, Zika NS1 For Primer: 5’-3329GCACAATGCCCCCACTGT3346-3’, Zika NS1 Probe: 5’-FAM-3349TTCCGGGCTAAAGATGGCTGTTGGT3373-TAMRA-3’, Zika NS1 Rev Primer: 5’-3394TGGGCCTTATCTCCATTCCA3375-3’ (sequence positions based on ZIKV Yap 2007 GenBank accession number EU545988) using assay conditions as previously described^10^. A pan-flavivirus heminested RT-PCR targeting the non-structural protein 5 gene^11^ was also performed.

Serological assessment of both acute and convalescent phase serum was performed using flavivirus IgG and IgM ELISAs and an in-house flavivirus IgM typing microsphere immunoassay (MIA) (C. Taylor, unpublished data). These assays utilise pooled and individual flavivirus antigens which include DENV 1-4, but exclude ZIKV. Patient serum was also tested using novel ZIKV IgG and IgM MIAs which incorporated recombinant ZIKV NS1 protein expressed in baculovirus (A. Pyke and P. Moore, unpublished data).

**Ethics Statement**

Ethical approval for this study was granted by the FSS Human Ethics Committee. The patient subject also provided written informed consent for disclosure and dissemination of clinical and laboratory findings.

## Results

**Molecular analysis**

The real-time RT-PCR assays targeting either the E or NS1 gene regions both detected ZIKV RNA in the acute patient serum sample with cycle thresholds of 29 and 28 respectively. This corresponded with positive RNA detection using the pan-flavivirus heminested assay. Additional nucleic acid sequencing of E and NS1 regions revealed 100% homology between patient ZIKV sequences and corresponding E and NS1 primer/probe TaqMan sequences. Although full validation of the E and NS1 ZIKV TaqMan assays is currently constrained due to limitations described above, specificities were assessed and no cross-reactivity with RNA from several other arboviruses was detected (Table 1).

**Table 1. Arboviruses used in this study.** 

Virus	Strain	Country	Source	Year of Isolation
Alfuy	MRM3926	Australia	Bird	1966
Kokobera	MRM32	Australia	Mosquito	1960
Kunjin	MRM16	Australia	Mosquito	1960
Murray Valley Encephalitis	MRM66	Australia	Mosquito	1960
Stratford	C338	Australia	Mosquito	1961
Yellow Fever	17D - vaccine strain derived from Asibi	Africa (Asibi)	Human (Asibi)	1937 (17D)
Japanese Encephalitis	TS00	Australia	Pig	2000
Dengue 1	ET00 234	Timor-Leste	Human	2000
Dengue 2	ET00 300	Timor-Leste	Human	2000
Dengue 3	ET00 209	Timor-Leste	Human	2000
Dengue 4	ET00 288	Timor-Leste	Human	2000
West Nile	NY99	Unites States of America	Bird	1999
Ross River	SV64	Australia	Mosquito	2007
Barmah Forest	BH2193	Australia	Mosquito	1974
Sindbis	MRM39	Australia	Mosquito	1960
Chikungunya	06113897	Mauritius	Human	2006

Further RT-PCR amplification and nucleic acid sequencing of the complete E gene revealed that the patient ZIKV strain CK-ISL 2014 (GenBank accession no KJ634273) was 99.5% homologous to previous Cambodian 2010 (GenBank accession number JN860885) and Yap Island 2007 (GenBank accession number EU545988) sequences and belonged to the Asian lineage (Figure 1).

**Maximum likelihood phylogenetic tree based on complete E gene nucleic acid sequence constructed using MEGA (www.megasoftware.net) with bootstrap support (1,000 replications).**
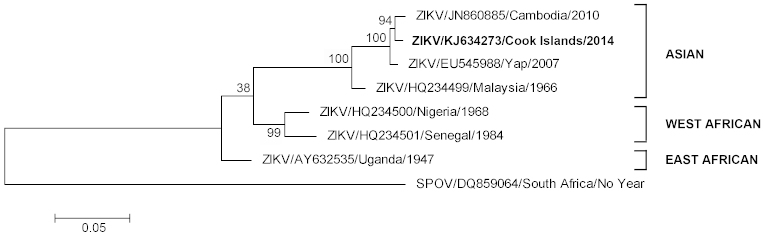

**Serological analysis**

Results of the flavivirus serological analyses indicated that the patient had previously been exposed to DENV, with detection of reactive flavivirus IgG and specific DENV-4 IgM antibodies in the acute sample. Similar results were obtained from the convalescent sample which demonstrated cross-reactive DENV 1-4 IgM antibodies. In contrast, seroconversions for both IgG and IgM were detected between the paired acute and convalescent sera using the respective ZIKV IgG and IgM MIAs.

## Discussion

With only three designated genotypes, West African (Senegal/Nigerian), East African (Uganda) or Asian, ZIKV is most closely related to Spondweni virus (SPOV)^4^. Due to limited availability of sequence data, the specific geographical origin(s) of the Yap, Cambodian or current Cook Island ZIKV strain(s) remains unknown; however it is most likely that introduction and sustained transmission of a Southeast Asian ZIKV strain(s) is responsible^3^, ^4^ and is causing widespread epidemics across the Pacific region.

Whilst DENV and CHIKV are not endemic in Australia, both viruses are regularly imported through viremic travelers, particularly from the Pacific, Papua New Guinea, Indonesia and other Southeast Asian countries. Within Australia, north Queensland and the Torres Strait are frequently susceptible to arbovirus transmission and human outbreaks due to the presence of suitable mosquito vectors and have historically experienced recurring DENV epidemics. The importation of yet another exotic arbovirus into north Queensland such as ZIKV underscores the region’s constant vulnerability to incursion and heightened risk of widespread disease. A popular tourist destination, north Queensland is in close geographical proximity to surrounding countries and locales experiencing ZIKV activity and rapid spread of the Asian lineage across the Pacific would indicate this strain is both genetically robust and highly sustainable in immunologically naïve populations. Should the Pacific ZIKV outbreak continue to spread unchecked or viral mutation(s) occur, similar to that of CHIKV in the Indian Ocean which increased virus transmission in the mosquito vector^12^, this could exacerbate both importation rates and case numbers. Together with the similar clinical syndrome and degree of cross-reactivity of patient ZIKV antibodies with related flaviviruses^1^^,^ 4 ^,^ 5, these factors necessitate a wider public health awareness for specifically identifying potential ZIKV cases and continued vigilance through accurate and rapid reporting, diagnosis and vector control.

In this short case report, we have described the first known identification and diagnosis of ZIKV infection in a patient returning to north Queensland. In the absence of ZIKV stocks and positive control patient samples, we were able to design and develop both molecular real-time RT-PCR and recombinant serological ZIKV assays in readiness prior to this importation. Although flavivirus serology strongly indicated a possible previous DENV infection, seroconversions of IgG and IgM were only demonstrated using the ZIKV recombinant NS1 MIAs. Interestingly, these findings from the recombinant MIAs were consistent with a recent ZIKV infection and correlated with molecular detection of ZIKV RNA in the acute phase sample.

Whilst further validation of the ZIKV real-time TaqMan and recombinant serology protocols is in progress, they have afforded rapid and potentially specific diagnosis of ZIKV infection in the patient who had traveled to the Cook Islands. This is not only relevant for laboratories with a high throughput of samples, but also emphasizes that pre-development of protocols for exotic viruses in the absence of viral or patient control material can be valuable to enhance capabilities and provide important and timely detection of future cases and outbreak events.

## Competing Interests

The authors have declared that there are no competing interests.

## Correspondence

Alyssa Pyke: Alyssa.Pyke@health.qld.gov.au
